# Establishment and characterization of an h*ACE2/*hTMPRSS2 knock-in mouse model to study SARS-CoV-2

**DOI:** 10.3389/fimmu.2024.1428711

**Published:** 2024-07-10

**Authors:** Hongwei Liu, Terza Brostoff, Ana Ramirez, Talia Wong, Douglas J. Rowland, Mollie Heffner, Arturo Flores, Brandon Willis, Jeffrey J. Evans, Louise Lanoue, K. C. Kent Lloyd, Lark L. Coffey

**Affiliations:** ^1^ Department of Pathology, Microbiology, and Immunology, School of Veterinary Medicine, University of California, Davis, CA, United States; ^2^ Center for Molecular and Genomic Imaging, College of Engineering, University of California, Davis, Davis, CA, United States; ^3^ Mouse Biology Program, University of California, Davis, Davis, CA, United States; ^4^ Department of Surgery, School of Medicine, University of California, Davis, Davis, CA, United States

**Keywords:** SARS-CoV-2, mouse ACE2, mouse TMPRSS2, knock-in mouse, COVID-19, virus, pathogenesis, pulmonary function

## Abstract

Despite a substantial body of research, we lack fundamental understanding of the pathophysiology of COVID-19 caused by severe acute respiratory syndrome coronavirus 2 (SARS-CoV-2) including pulmonary and cardiovascular outcomes, in part due to limitations of murine models. Most models use transgenic mice (K18) that express the human (h) angiotensin converting enzyme 2 (*ACE2*), *ACE2* knock-in (KI) mice, or mouse-adapted strains of SARS-CoV-2. Further, many SARS-CoV-2 variants produce fatal neurologic disease in K18 mice and most murine studies focus only on acute disease in the first 14 days post inoculation (dpi). To better enable understanding of both acute (<14 dpi) and post-acute (>14 dpi) infection phases, we describe the development and characterization of a novel non-lethal KI mouse that expresses both the *ACE2* and transmembrane serine protease 2 (*TMPRSS2*) genes (h*ACE2*/h*TMPRSS2*). The human genes were engineered to replace the orthologous mouse gene loci but remain under control of their respective murine promoters, resulting in expression of *ACE2* and *TMPRSS2* instead of their murine counterparts. After intranasal inoculation with an omicron strain of SARS-CoV-2, h*ACE2*/h*TMPRSS2* KI mice transiently lost weight but recovered by 7 dpi. Infectious SARS-CoV-2 was detected in nasopharyngeal swabs 1-2 dpi and in lung tissues 2-6 dpi, peaking 4 dpi. These outcomes were similar to those in K18 mice that were inoculated in parallel. To determine the extent to which h*ACE2/*h*TMPRSS2* KI mice are suitable to model pulmonary and cardiovascular outcomes, physiological assessments measuring locomotion, behavior and reflexes, biomonitoring to measure cardiac activity and respiration, and micro computed tomography to assess lung function were conducted frequently to 6 months post inoculation. Male but not female SARS-CoV-2 inoculated h*ACE2/*h*TMPRSS2* KI mice showed a transient reduction in locomotion compared to control saline treated mice. No significant changes in respiration, oxygen saturation, heart rate variability, or conductivity were detected in SARS-CoV-2 inoculated mice of either sex. When re-inoculated 6 months after the first inoculation, h*ACE2/*h*TMPRSS2* KI became re-infected with disease signs similar to after the first inoculation. Together these data show that a newly generated h*ACE2/*h*TMPRSS2* KI mouse can be used to study mild COVID-19.

## 1 Introduction

The coronavirus disease 2019 (COVID-19) pandemic caused by severe acute respiratory syndrome coronavirus 2 (SARS-CoV-2) continues to cause a massive global burden with excess mortality and disruptions to social, economic, and healthcare systems. COVID-19 produces diverse manifestations that can affect respiratory, cardiovascular, neurological, immunological, and gastrointestinal systems (reviewed in (1, 2)). Many infected people report shortness of breath, fatigue, and exercise intolerance (1, 2) which are likely due to cardiovascular and pulmonary involvement. After SARS-CoV-2 infects the respiratory tract, inflammation can lead to irreversible pulmonary fibrosis and bronchiectasis that ultimately compromise respiratory function (3–8). Chest pain, dyspnea and heart palpitations, as well as more severe cardiovascular disease may result in inflammation, myocardial infarction, and other cardiac manifestations (reviewed in (2)). The underlying pathophysiological mechanisms for both respiratory and cardiovascular COVID-19 manifestations are still poorly understood. This lack of understanding in part stems from limitations inherent to human studies, where invasive assessments, repeated sampling, and human tissues are often not available. To circumvent these limitations, animal models of COVID-19 represent a valuable complement to information learned from human studies.

Animal models have been extensively used to study pathogenesis of acute SARS-CoV-2 infection and for evaluating COVID-19 countermeasures including vaccines and therapeutics (reviewed in (9)). Syrian hamsters typically recover from weight loss and interstitial pneumonia after SARS-CoV-2 infection, but are not widely used because of their size, aggressiveness, cost to maintain, and unfamiliar biology and husbandry requirements (9–12). Mice are especially valuable as COVID-19 models due to their rapid breeding, gestation, growth rates, highly characterized immune systems, and ease of genetic manipulation. Common murine models of acute COVID-19 include wildtype mice inoculated with serial mouse passaged SARS-CoV-2, engineered mice that express the human angiotensin converting enzyme 2 (*ACE2*) receptor under control of the cytokeratin-18 (Krt18 (K18)) promoter for epithelial cell expression, or mice that transiently express *ACE2* via adenovirus or adeno-associated virus (AAV) expression systems (reviewed in (9)). Depending on the murine model, virus variant, and dose employed, SARS-CoV-2 inoculated mice variously develop weight loss, detectable viral RNA and infectious virus in the respiratory tract, lung inflammation and injury, and death. Some models also show extrapulmonary SARS-CoV-2 detection in selected tissues including the brain which corresponds with neurologic signs of disease, likely due to non-physiological expression of h*ACE2* in the brain. While these models collectively serve as an extremely valuable resource for development of therapies and prophylactic measures, further improvements to murine models can enable understanding of the pathophysiological consequences of acute infection relevant to humans. Further, most experimental endpoints for murine studies using extant models do not exceed 2 weeks, which occurs before the onset of long COVID-19.

To model human expression of *ACE2* and thereby avoid neuroinvasive SARS-CoV-2 and neurologic or lethal disease characteristic of pre-omicron variants in the K18 model, we sought to develop a new mouse model. We reasoned that mice expressing human orthologous genes known to be involved in virus binding, entry, and activation similar to those in humans would better represent COVID-19. Our approach involves incorporating the human coding sequence directly into the endogenous mouse locus, thus replacing the orthologous mouse genes while recapitulating the expression level, pattern, and timing of the human gene under the control of specific neighboring gene regulators. This dual humanized (h) knockin/mouse knockout approach overcomes inherent genetic discrepancies between mouse and human genes, is highly relevant to human biology and disease compared to random genomic integration of multiple copies of coding sequence as seen in transgenic mice and can be used to generate murine models that are more efficiently managed and more cost effective than hamsters. Our approach is also predicated on the success of related *ACE2* knock-in (KI) models (13–16) in which the *Ace2* gene was substituted with *ACE2*. For example, in Winkler et al. (14), h*ACE2* KI mice express *ACE2* in lung, nasal turbinate, kidney, duodenum, and olfactory bulb, but not in colon, ileum, heart, spleen, or liver. After challenge with an early 2020 SARS-CoV-2 strain, viral infection was mainly restricted to the murine respiratory tract but not extrapulmonary tissues (including brain), implying an absence of *ACE2* expression in the brain (which was not shown). Mice experienced no weight loss and limited histopathologic changes in the lung 3 days post-inoculation (dpi). These data demonstrate susceptibility of *hACE2* KI mice to SARS-CoV-2. Building on these established *hACE2* KI models, we also predicted that expression of an additional human gene involved in SARS-CoV-2 infection may more closely recapitulate human COVID-19. We therefore generated KI mice expressing both *ACE2* and human transmembrane serine protease 2 (*TMPRSS2*), where *TMPRSS2* encodes a protease that cleaves the SARS-CoV-2 spike protein after binding to *ACE2* to facilitate virus invasion and activation (17). After generating and validating expression of the human genes in these novel h*ACE2/*h*TMPRSS2* KI mice, we characterized acute SARS-CoV-2 infection dynamics and long-term changes in physiology, behavior, cardiac and respiratory activity, and lung function to evaluate suitability of the model for COVID-19. Our data show that h*ACE2/*h*TMPRSS2* KI survive SARS-CoV-2 infection, and male but not female mice develop transient reductions in locomotion like COVID-19 affected humans. New polygenic humanized models like the h*ACE2/*h*TMPRSS2* KI mouse developed here can provide new knowledge regarding SARS-CoV-2 infection and COVID-19 in humans and enable experiments to observe, monitor, and assess COVID-19 outcomes with current and emerging variants.

## 2 Materials and methods

### 2.1 Ethics statement

All mouse work was conducted on protocol #23489 approved by the institutional animal care and use committee (IACUC) at the University of California, Davis. Infectious virus was handled in certified animal biosafety level 3 laboratory (ABSL-3) spaces in compliance with approved institutional biological use authorization #R2813. The University of California, Davis, is accredited by the Association for Assessment and Accreditation of Laboratory Animal Care (AAALAC). All mouse work adhered to the NIH Guide for the Care and Use of Laboratory Animals.

### 2.2 Generation of humanized *ACE2*, *TMPRSS2*, and *ACE2/TMPRSS2* knock in mice

Human *ACE2* and *TMPRSS2* KI alleles were designed to replace expression of the endogenous mouse *Ace2* and *Tmprss2* genes with the corresponding full length human coding sequence (CDS) under transcriptional control by mouse regulatory elements. CRISPR Cas9 was used to target murine embryonic stem (ES) cells using a single guide RNA (gRNA) complexed with Cas9 nuclease as a ribonucleic protein (RNP) in the presence of a plasmid repair template harboring the coding sequence for both h*ACE2* and h*TMPRSS2* and mouse 3’ untranslated region (UTR) flanked by 1 kilobase (kb) homology arms. Guide RNAs were screened with an established approach (18) and selected using the publicly available tool http://crispor.tefor.net/crispor.py.

*TMPRSS2* plasmid repair template was synthesized as a gene product (IDT, Coralville, IA) and *ACE2* was constructed using Gibson assembly with synthesized G-block fragments (19). Once genetically verified by sequencing, plasmids were propagated and purified using an EndoFree Plasmid Maxi Kit following manufacturer’s instruction (Qiagen, Germantown, MD) and subsequently validated by restriction fragment analysis followed by full sequencing of critical regions. RNP assembly included pairing of trans-acting CRISPR RNA (tracrRNA) with CRISPR RNA (crRNA) with a 2 minute denaturation at 95°C followed by room temperature incubation for 5 minutes to produce the guide RNA (gRNA) and then complexed with s.p. Cas9 Nuclease V3 for 10 minutes at 37°C at a 1.2:1 molar ratio (IDT). Constructed circular repair template plasmids along with a PGK-neomycin containing plasmid PGKneoF2L2DTA (Addgene, Watertown, MA, #13445) for transient selection were added for a final concentration of 2.4ug/uL RNP, 0.4ug/uL repair template plasmid, and 0.2ug/uL of neomycin plasmid. CRISPR targeting reagents were electroporated into JM8A3 C57BL/6N Agouti ES cells (20) on a BTX ECM 630 Pulse Generator set at 700V, 400Ω, 25µF and selected for 2 to 3 days with 400 ug/uL G418 following standard culture protocols (21). Individual ES cell colonies were picked and submitted for genetic analysis with KI copy number, 5’ and 3’ long range PCR, sequence confirmation, and pathogen free status confirmed prior to blastocyst injection. Genetically confirmed clones containing a single copy of the expected KI alleles were expanded for injection into C57BL/6N blastocysts to obtain high percentage chimeric F0 mice. Genetically-confirmed chimeras were backcrossed to C57BL/6N to produce first generation N1 progeny which were genetically screened by PCR for germline transmission of the KI allele (20). PCR positive N1 animals were then verified for correct targeting and copy number of the allele (**Supplementary Figure S1**). h*ACE2* and h*TMPRSS2* KI mice were then intercrossed to create a true breeding bi-genic h*ACE2/*h*TMPRSS2* KI line for propagation and experimental virus inoculation and analyses.

### 2.3 Characterization of gene expression in *hACE2/hTMPRSS2* knock-in mice

Approximately 10 mg of lung, upper respiratory tract (URT), brain, and lower intestine were collected from 2 females and 2 males of h*ACE2*/h*TMPRSS2* KI, as well as age- and sex- matched control (Wildtype) mice. Tissues were placed into RNAlater and stored at 4°C overnight. RNA was subsequently extracted using the RNeasy Mini Kit with on column DNase treatment following manufactures protocol (Qiagen). Purified RNA was analyzed by NanoDrop for concentration and purity and visualized on an agarose gel for confirmation of integrity. Each RNA sample was normalized for concentration and then converted to cDNA using the High-Capacity cDNA Reverse Transcription Kit according to the manufacturer’s protocol (ThermoFisher). cDNA samples were processed in triplicate using the TaqMan™ Gene Expression Master Mix (ThermoFisher) for each target with sequence specific oligos (**Supplementary Table 1**) and multiplexed with a stable housekeeping gene (*Actb*) using TaqMan assays with 6FAM reporter per target and VIC reporter for endogenous reference (IDT, ThermoFisher). 384 well plates were processed on a QuantStudio7 and analyzed with QuantStudio™ software using the relative Ct method (ΔΔCt) (22). Data shown represent values measured from all animals.

### 2.4 K18 human ACE2 transgenic mice

Transgenic mice (B6.Cg-Tg(K18-ACE2)2Prlmn/J; RRID : IMSR_JAX:034860) expressing the human ACE2 gene (K18) were obtained from The Jackson Laboratory (Strain #034860, Bar Harbor, ME) and maintained under identical vivarium conditions as h*ACE2/*h*TMPRSS2* KI mice.

### 2.5 Cell lines, SARS-CoV-2 isolates, and heat inactivation of SARS-CoV-2

African Green monkey kidney epithelial cells (Vero-E6, NR-53728), Vero E6 expressing *ACE2* and *TMPRSS2* (Vero-E6-TMPRSS2-T2A-ACE2, NR-54970) for culturing omicron, and Vero CCL-81 were obtained from ATCC/BEI (Manassas, VA). Vero-E6 and Vero CCL-81 cells were cultured at 37°C with 5% CO_2_ in Dulbecco’s modified Eagle medium (DMEM; Gibco, ThermoFisher Scientific, Emeryville, CA) supplemented with 5% fetal bovine serum (FBS; Genesee Scientific, San Diego, CA) and 1x antibiotic-antimycotic (Fisher Scientific, Waltham, MA). Vero-E6-TMPRSS2-T2A-ACE2 cells were cultured under the same conditions as the other Vero cell lines with the addition of 10 µg per mL puromycin (Fisher Scientific, Waltham, MA). SARS-CoV-2 strains were propagated one additional time after procurement in Vero CCL-81 cells to achieve titers of 6-7 log_10_ Vero plaque forming units (PFU)/mL, respectively. Single use virus aliquots were stored at -80°C until thaw for use in murine experiments. For studies with heat inactivated SARS-CoV-2, 5 log_10_ PFU of SARS-CoV-2 B.1.1.529 (omicron) diluted in Dulbecco’s phosphate buffered saline (DPBS) was split into 2 tubes, one of which was subjected to heat treatment in a water bath at 56°C for 30 minutes. The other tube was held at 4°C during heat inactivation. The absence of detectable infectivity in the heat inactivated tube was verified by titration. Both tubes were used for inoculation into mice.

### 2.6 SARS-CoV-2 mouse inoculation and re-inoculation

h*ACE2/*h*TMPRSS2* KI mice aged 12 weeks and 7-12 week old K18 mice of both sexes were weighed and anesthetized with isoflurane, then inoculated intranasally (i.n.) via hanging drop over both nares with 30 µl DPBS, 5 log_10_ PFU of SARS-CoV-2 B.1.1.529 (omicron) diluted in DPBS, or 5 log_10_ PFU of heat inactivated SARS-CoV-2 B.1.1.59 diluted in DPBS. Before settling on omicron, we inoculated 4 log_10_ PFU of different strains (**Table 1**) of SARS-CoV-2 into K18 mice. These included B.1, alpha, beta, delta, and 2 doses (4 log_10_ and 5 log_10_ PFU) of the omicron B.1.1.529 strain. To evaluate susceptibility and disease signs after a second infection, a subset of h*ACE2/*h*TMPRSS2* KI mice of both sexes that had been inoculated first at 12 weeks of age with 5 log_10_ PFU omicron were re-inoculated 182 days (6 months) later, at 38 weeks of age, with 5 log_10_ PFU of the same omicron strain. Inocula were back-titrated to confirm the target dose. Mice were monitored once daily for changes in weight, ruffled fur, ataxia, and labored breathing for up to 15 days. On days 1 and 2 post-inoculation (dpi), mice were anesthetized with isoflurane and oropharyngeal samples were obtained by swabbing with rayon-tipped swabs (Puritan, Fisher Scientific, Fisher Scientific, Waltham, MA). Swabs were vortexed briefly in 500 μL of DMEM containing 1% FBS and frozen at -80°C. A subset of mice were euthanized on 2, 4, or 6 dpi and the remaining animals were euthanized at experiment end. At 17, 67, or 199 dpi or prior to euthanasia, blood was collected by submandibular vein puncture under isoflurane anesthesia. Whole blood was clotted for >10 minutes at room temperature then centrifuged for 5 minutes at 8,000 x g and cleared serum was stored at -80°C. Mice were euthanized by isoflurane overdose and cervical dislocation then perfused with sterile DPBS. The right inferior lung lobe and the left hemisphere of the brain were weighed and homogenized in 500 μL DMEM with a sterile glass bead at 30 Hz for 4 minutes using a TissueLyser (Qiagen, Germantown, MD). Homogenates were cleared by centrifugation at 10,000 x *g* for 4 minutes and the cleared fraction was stored at -80°C.

**Table 1:** SARS-CoV-2 strains used in mouse experiments.

Strain	Variant	Lineage	Location of Provider	Source	Passage	Viral titer Vero PFU/ml
**hu/USA/CA-CZB-59X002/2020. (MT394529)**	WA1-like	B.1	CA, USA	UC Davis Center for Immunology and Infectious Diseases	p2	2.2x10^7^
**hu/USA/CA_CDC_5574/2020**	Alpha	B.1.1.7	CA, USA (UK origin)	BEI, NR-54011, batch: 70041598	p1	7.7x10^6^
**hCoV-19/USA/MD-HP01542/2021**	Beta	B.1.351	USA (African origin)	BEI, NR-55282, batch: 70043066	p1	2x10^7^
**hCoV-19/USA/PHC658/2021**	Delta	B.1.617.2	USA (India origin)	BEI, NR-55611, batch: 70045238	p1	1.1x10^7^
**hCoV-19/USA/HI-CDC-4359259-001/2021**	Omicron	B.1.1.529	USA	BEI, NR-56475, Batch: 70049691	p1	2.1x10^7^
**hCoV-19/USA/MD-HP40900/2022 (XBB.1.5),**	Omicron	XBB.1.5	USA	BEI, NR-59104, batch: 70057837	p1	7.2x10^7^

### 2.7 SARS-CoV-2 titrations

Fluid collected from oropharyngeal swabs, residual inocula, and lung and brain homogenates were thawed and assayed to quantify infectious SARS-CoV-2. Undiluted (125 μl) and serial 10-fold diluted samples in DMEM with 1% FBS were inoculated into confluent Vero-E6 or Vero-E6-TMPRSS2-T2A-ACE2 (used for omicron only) cells in 12-well plates with cell culture media decanted. Cells were incubated for 1 hour at 5% CO_2_ in a humidified 37°C chamber. After the incubation, cell monolayers were overlaid with 0.5% agarose dissolved in DMEM with 5% FBS and 1x antibiotic-antimycotic and held for 2 (for omicron only) or 3 (all other viruses) days at 5% CO_2_ and 37°C in a humidified incubator. After 2 or 3 days, cells were fixed for >30 minutes with 4% formaldehyde then agarose plugs were removed. Cells were stained with 0.05% crystal violet in 20% ethanol for 10 minutes then rinsed 3 times with water. Plates were inverted to dry completely and the number of plaques in each well was counted. Viral titers were recorded as the reciprocal of the highest dilution where plaques were noted and are represented as PFU per mL inoculum, swab, or mg of tissue. Each value reported represents a titer calculated from 1 replicate titration of serial dilutions. The lower limit of detection (LOD) of the assay was 8 PFU/ml. Samples with no detectable plaques are reported at the LOD.

### 2.8 Neutralizing antibody assessments

For plaque reduction neutralization test 50% (PRNT_50_) to quantify neutralizing antibody titers, serum from blood collected on 17, 67 or 199 dpi (where the last time is 18 days after the second inoculation) was thawed at 37°C and 40 μL was heated to 56°C in a water bath for 30 minutes to inactivate complement proteins. Serum was diluted 5-fold in PBS and 1% FBS, then samples were serially 2-fold diluted 10 times for a dynamic range of 1:5 to 1:2560. An equal volume of virus diluent containing 80 PFU of SARS-CoV-2 homologous to the inoculum strain was added to each antibody dilution and a no-antibody control consisting of virus diluent only, resulting in a final dynamic range of 1:10 to 1:5126. Antibody-virus dilution series were incubated for 1 hour at 37°C after which they were applied to confluent Vero-E6 cells in single replicates and incubated for 1 hour at 5% CO_2_ and 37°C in a humidified incubator. Cells were overlaid, incubated, fixed, and stained as described above for plaque assays. The neutralization titer was defined as the reciprocal of the dilution for which fewer than 50% of plaques were detected versus the no-antibody control (>50% neutralization). Each value reported represents a titer calculated from 1 replicate PRNT_50_ assay.

### 2.9 Behavioral, respiratory, and cardiac assessments

Mice inoculated with SARS-CoV-2 or DPBS were assessed for locomotion, behavior, reflexes, cardiac activity, respiration, and lung morphology and function 5 days prior to inoculation and again 3 or 4, 15 or 16, and 30 or 31 dpi, then monthly thereafter within a 2 day window ending on 150 or 151 dpi. Phenotyping was performed on consecutive days, where the order, time of day, environment, and experimenters throughout the testing period were consistent. Experimenters were not blinded to SARS-CoV-2 or DPBS treatment for biosafety reasons, although all mice were housed in the ABSL-3 and handled identically. Assessments of morphology and gross motor and sensory functions were performed using a modified SmithKline Beecham, Harwell, Imperial College, Royal London Hospital, phenotype assessment (SHIRPA) primary screen test that includes a battery of observational measures of behaviors and functional assessments, implemented according to an International Mouse Phenotyping Consortium protocol (23). After a 15 minute acclimation period to testing room environmental conditions, mice were placed in a viewing jar to observe general appearance (e.g., coat, whiskers, teeth, and eyes), and behaviors (e.g., body position, tremor, head bobbing). Afterwards, mice were placed into an arena from a height of approximately 30 cm to assess transfer arousal, locomotor activity, gait, tail elevation, touch escape and startle response. Transfer arousal was measured as the delay (in seconds) before the mouse began to explore the arena. Locomotor activity was measured by tallying the number of squares crossed by the mouse in the first 30 seconds. Gait and tail elevation were observed during ambulation of the mouse in the arena. Touch escape was evaluated by the fleet response to an extended index finger; mouse should flee prior physical contact. Startle was evaluated using a click box activated behind the mouse. Finally, mice were manipulated to evaluate positional passivity, trunk curl, limb grasping, and contact righting reflex, and given a complete physical exam. Mice were placed in either a viewing jar or a larger arena. Observations included muscle, cerebellar, sensory, and autonomic functions as well as morphology. Overall, more than 40 specific behaviors and morphological parameters were assessed by observation and scored as present/absent or as expected/abnormal. Cardiac electrophysiology was evaluated by electrocardiogram (ECG) on anesthetized mice. Mice were anesthetized with 5% isoflurane and transferred to a warm rodent surgical monitor platform (Scintica, London, Canada) in a prone position with a nose cone set a 2.5% isoflurane and a rectal probe to monitor body temperature. Hindlimb feet were secured to surface electrodes on the platform and ECG signals were recorded for 2 minutes. A section of the ECG tracing was selected and analyzed using the LabScribe software (iWorx, Dover, NH). P, Q, R, S and T peaks in ECG were automatically detected by the software and peak intervals and amplitudes were averaged over several cardiac cycles. Heart rate and heart rate variability were averaged over the entire length of the ECG tracing. Respiration rates and arterial blood oxygen saturation were recorded over 2 minutes using a pulse oximeter sensor (Scintica) secured to the right leg of mice and are reported as the mean from all mice in the cohort. All cardiorespiratory parameters were analyzed, and respiration rates (respiration per minute, rpm), QT interval corrected for heart rate (QTc) and heart rate variability (dRR) are reported herein. Data are boxplots with means and standard deviation, where each dot shows data from 1 measurement from 1 mouse.

### 2.10 Micro computed tomography and lung function assessments

Respiratory gated lung imaging by micro (µ)CT was performed under inhalation anesthesia. Male and female mice that were SARS-CoV-2 or DPBS inoculated were anesthetized with 5% isoflurane and transferred to a µCT imager. To maintain primary biosafety containment of animals upon transfer to the µCT, a custom imaging chamber was designed and built (UC Davis TEAM Facility) to provide HEPA air filtration of delivered anesthetic gas during imaging. Animals were maintained on 1-1.5% isoflurane during imaging with the Quantum GX2 µCT (Revvity, Waltham, MA). Images were acquired in respiratory gating mode using a Cu/Al filter, 90 kV, and 88 µA. Images were reconstructed into end inspiration and end expiration volumes at 72 micrometer voxel size. Imaging was performed multiple times on each mouse at -6, 5, 17, 33, 58, 94, 128 and 158 dpi. For the re-inoculation study, mice were imaged 6 days before and 5 and 17 days after the second inoculation. Pulmonary changes consistent with SARS-CoV-2 infection were evaluated by visual inspection and by segmenting aerated lung tissue. Whole lung segmentation was performed using a Sensor 3D deep learning model (24). The model was trained on an AMD Ryzen Threadripper Pro 3955WX workstation with a GeForce RTX 3090 Ti graphics card. The model was implemented in Dragonfly 2022.2 and had 15 time distributed convolutions (activation function = Conv2D), four time-distributed max pooling, and 2 bidirectional convolution long-short term memory steps. The model was trained with a patch size of 64, stride ratio 0.5, batch size 32, a loss function looking at the categorical cross entropy, and optimized with the Adadelta function with a linear learning rate set to 1. Data augmentation x10 was implemented with flip, rotation, scaling, brightness and gaussian noise. An image mask (347 x 347 x 277) was applied to 6 training sets (3 end inspiration and 3 end expiration scans) to minimize training time, which took approximately 82 hours. The model was applied to 6 additional test datasets. The model computation time for additional scans is under 2 minutes and requires minimal processing. The segmented lung was further split into aerated lung and connective/vessel tissues by Otsu’s method (25).

### 2.11 Histopathologic assessments

For necropsy, a lungs from 4 SARS-CoV-2 and 4 DPBS-inoculated h*ACE2/*h*TMPRSS2* KI and K18 mice were inflated with 10% buffered formalin (Fisher Scientific, Waltham, MA) followed by fixation for 48 hours at room temperature in an approximate 10-fold volume of formalin. Lungs were then embedded in paraffin, thin-sectioned, and stained with hematoxylin and eosin (H&E). H&E-stained slides were scanned by a whole-slide image technique using an Aperio slide scanner (Leica, Buffalo Grove, IL) with a resolution of 0.24 μm/pixel. Image files were uploaded on a Leica hosted web-based site and a board certified veterinary anatomic pathologist without knowledge of treatment conditions evaluated sections for SARS-CoV-2 induced histologic lesions. A first round of scoring was conducted while the pathologist was masked to phenotype, and a second round was conducted after unmasking to allow comparison between DPBS- and SARS-CoV-2 inoculated mice, as well as between *hACE2/TMPRSS2* KI and K18 mice. The histopathologic grading scheme was developed with the goal of establishing a comprehensive scoring rubric that could accurately and reproducibly characterize the severity of pulmonary lesions specific to SARS-CoV-2. Each lung was scored using a rubric we previously described (26). Scores reported are a single composite measurement from evaluation of the entire lung for each mouse. Scores range from 0 (no significant findings) to 4 (severe lesions involving >25% of the pulmonary parenchyma), with an additional point possible for >25% neutrophilic inflammation, necrotizing vasculitis, and/or microthrombi.

### 2.12 Statistical analyses

Statistical assessments for were performed using SAS (SAS, version 9.4, SAS Institute, Cary, NC) or GraphPad Prism version 10 (Boston, MA). Repeated measures two-way ANOVA tests were performed on log_10_ transformed SARS-CoV2 titers and multiple comparisons were computed according to Tukey method. Main effect two-way ANOVA tests were performed on mouse weights normalized to 0 day values at the time of virus inoculation and on log_10_ transformed viral titers in tissues and multiple comparisons were computed with Tukey’s method. Differences in locomotion and lung function parameters in males or females over time were analyzed using a 2-way analysis of variance (ANOVA) with time, treatment and the interaction of time and treatment as main effects. Differences in respiration, heart rates and ECG markers over time and across the SARS-CoV-2 or DPBS inoculated h*ACE2*/h*TMPRSS2* KI mice were analyzed by analysis of variance using a linear mixed model for each sex. Histopathologic lung scores were compared using two-way ANOVA with multiple comparisons.

## 3 Results

### 3.1 Generation of and characterization of gene expression in h*ACE2/*h*TMPRSS2* knock-in mice

**Figure 1:**
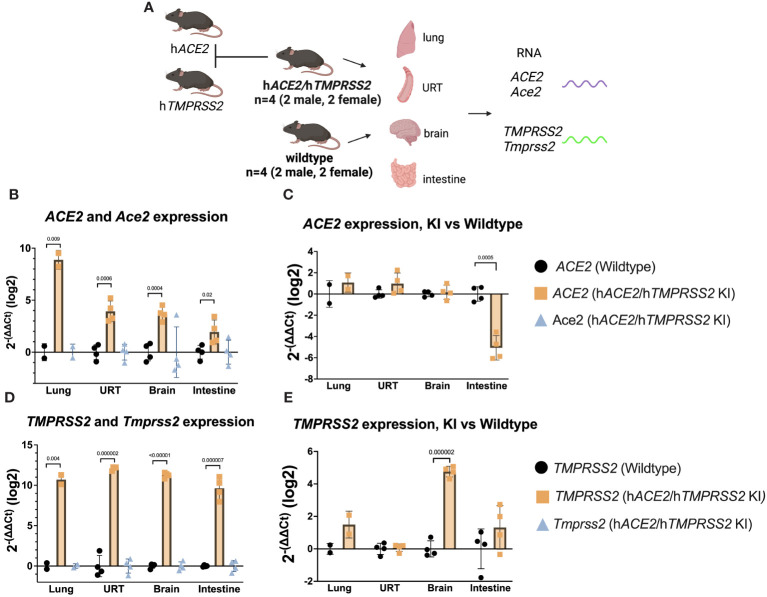
Transcript expression levels of *ACE2* and *TMPRSS2* in comparison to endogenous mouse orthologs *Ace2* and *Tmprss2*. **(A)** Schematic of mice and tissues for gene expression to validate mouse models. **(B-E)** Upper respiratory tract (URT), brain, and intestine were harvested from 4 (2M/2F) h*ACE2*/h*TMPRSS2* KI mice and lung harvested from 2 (1M/1F) h*ACE2*/h*TMPRSS2* KI mice as well as from the same number of age- and sex- matched Wildtype mice. qPCR using TaqMan probes from RNA derived cDNA for each human and mouse target was performed and values were compared to an *Actb* reference using the relative Ct method (ΔCt) in triplicate. Averaged ΔCt per sample and human or mouse assay were used to generate 2^-(ΔΔCt)^ for each sample as compared to the averaged Wildtype control group and transformed to log_2_. Each dot shows the measurement from one animal and bars show group means. P values were generated using t-tests and shown above bars; groups lacking p values were not significantly different at p<0.05.

*ACE2* and *TMPRSS2* expression were measured in tissues collected from h*ACE2*/h*TMPRSS2* KI mice and compared to levels in tissues harvested from control Wildtype mice (**Figure 1A**). *ACE*2 transcript expression, represented as log^2^ of 2^-(ΔΔCt)^ above Wildtype background, was detected in lung, upper respiratory tract (URT), brain, and intestine of h*ACE2/*h*TMPRSS2* KI mice, but not in Wildtype mice (**Figure 1B**). Similarly, *TMPRSS2* transcript was detected in all tissues tested in h*ACE2*/h*TMPRSS2* KI mice but not in Wildtype mice. As expected, neither *Ace2* (**Figure 1B**) or *Tmprss2* (**Figure 1D**) orthologs were expressed in h*ACE2*/h*TMPRSS2* KI mice, confirming that genomic insertion of *ACE2* and *TMPRSS2* coding sequences into the cognate mouse loci effectively interrupted and blocked expression of the corresponding mouse gene orthologs. To further assess relative expression of human and mouse genes, *ACE2* and *TMPRSS2* ΔCt in h*ACE2*/h*TMPRSS2* KI mice were compared to *Ace2* and *Tmprss2* ΔCt in Wildtype mice, respectively. There was no statistically significant difference in levels of expression between *ACE2* and *Ace2* in lung, URT, or brain, although *ACE2* expression was significantly less than *Ace2* in the intestine (**Figure 1C**). Similarly, there was no statistically significant difference in level of expression between *TMPRSS2* and *Tmprss2* in lung, URT, or lower intestine, although *TMPRSS2* expression was significantly higher than *Tmprss2* in brain (**Figure 1E**).

### 3.2 SARS-CoV-2 kinetics and tropism in knock-in mice

**Figure 2:**
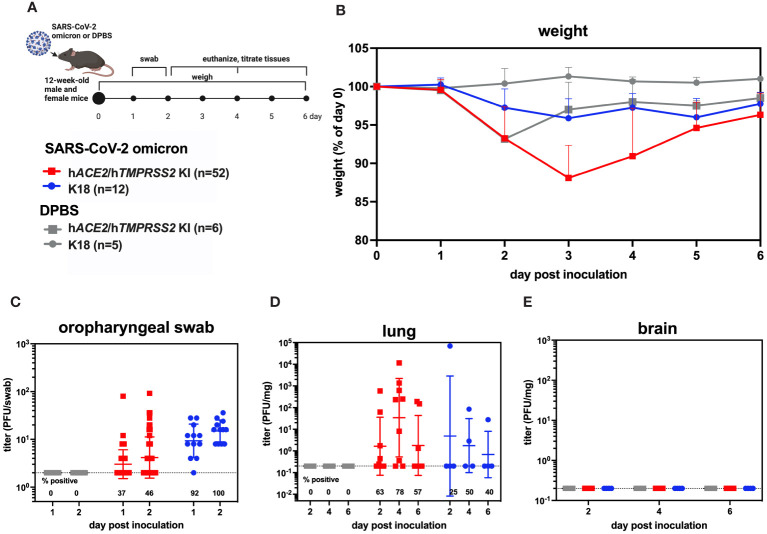
SARS-CoV-2 B.1.1529 (omicron) infection kinetics in h*ACE2/*h*TMPRSS2* KI and K18 mice. **(A)** Twelve-week-old male and female KI or K18 mice were inoculated intranasally with DPBS or 5 log_10_ PFU SARS-CoV-2 B.1.1.529 (omicron). Mice were monitored and weighed daily and the oropharynx was swabbed on 1 and 2 dpi. A subset of mice were euthanized at 2, 4, and 6 dpi. **(B)** Mean body weight change represented as a percentage of weight at the time of inoculation. Each timepoint represents mean with standard deviation. A main effect only model two-way ANOVA with Tukey corrected multiple comparisons yielded p<0.0001. Infectious SARS-CoV-2 titrated from **(C)** oropharyngeal swabs collected 1 and 2 dpi, and from necropsy samples in **(D)** lung and **(E)** brain on 2, 4, and 6 dpi, as quantified by plaque assay. Symbols represent individual animals, horizontal lines represent geometric mean, and error bars represent geometric standard deviation. Numbers below the dotted limit of detection lines indicate the percentage of mice with a detectable SARS-CoV-2 titer. Mean titers between groups of mice sampled on the same day were not significantly different from each other (paired T-tests on log-transformed PFU titers, p>0.05). Data shown are combined from three replicate experiments.

To understand SARS-CoV-2 infection and tropism in the KI mice, twelve-week-old male and female h*ACE2/*h*TMPRSS2* KI were inoculated intranasally (i.n.) with DPBS or 5 log_10_ PFU of SARS-CoV-2 B.1.1.529, which is an omicron variant (**Figure 2A**). Transgenic B6.Cg-Tg(K18-*ACE2*)2Prlmn/J mice expressing h*ACE2*, hereafter termed K18 mice, were used as a comparator. Weight compared to day 0 was measured once daily post-inoculation for each mouse and is represented as the mean of all animals. Starting 1 dpi, K18 mice inoculated with SARS-CoV-2 developed and sustained mean weight loss of about 5%, which contrasts with K18 mice administered DPBS, who maintained near 100% of 0 day weights (**Figure 2B**). By contrast, h*ACE2/*h*TMPRSS2* KI mice inoculated with DPBS or SARS-CoV-2 experienced a transient mean weight loss of about 7% beginning 2 dpi. Since both groups of h*ACE2/*h*TMPRSS2* KI mice were anesthetized to enable oropharyngeal swabbing 1 and 2 dpi, we attribute this weight loss to anesthesia or swabbing and infer that the h*ACE2/*h*TMPRSS2* KI mice are more susceptible to these effects compared to DPBS treated K18 mice, which did not show weight loss after the same procedures. Even with these treatment related changes in weight, mean weight loss in SARS-CoV-2 inoculated h*ACE2/*h*TMPRSS2* KI mice was significantly more than in DPBS treated h*ACE2/*h*TMPRSS2* KI (two-way ANOVA with multiple comparisons, p<0.0001), suggesting that some weight loss we observed was virus induced. At 2 dpi, infectious SARS-CoV-2 was detected in oropharyngeal swabs from 46% of h*ACE2/*h*TMPRSS2* KI mice, compared to 100% of K18 mice (**Figure 2C**). The mean swab titer in SARS-CoV-2 inoculated h*ACE2/*h*TMPRSS2* KI mice 1 or 2 dpi did not significantly differ; similarly, K18 mice showed no difference in mean swab titer on 1 or 2 dpi (paired t-tests, p>0.05). Infectious SARS-CoV-2 was detected in lungs of most SARS-CoV-2 inoculated h*ACE2/*h*TMPRSS2* KI mice on 2, 4, or 6 dpi, with titers ranging from the limit of detection of 0.2 PFU/mg to more than 4 log_10_ PFU/mg lung (**Figure 2D**). The mean lung titer on each day or between h*ACE2/*h*TMPRSS2* KI and K18 was not significantly different (paired t-test, p>0.05). Infectious virus was detected in at least 1 swab or the lungs for most of h*ACE2/*h*TMPRSS2* KI mice euthanized 2, 4, or 6 dpi, which was our criteria for demonstrating infection. However, given that not all mice met this metric, to ensure that titrated virus in murine samples did not reflect non-replicating residual inoculum, we also performed an experiment wherein cohorts of h*ACE2/*h*TMPRSS2* KI mice were inoculated with 5 log_10_ PFU SARS-CoV-2 omicron that had been heat inactivated to ablate infectivity (**Supplementary Figure S2A**). The same dose of virus and strain that was not heat inactivated was used as a comparator. No mouse inoculated with heat-inactivated SARS-CoV-2 omicron had detectable infectious virus in oropharyngeal swabs at 2 or 3 dpi (**Supplementary Figure S2B**), or in trachea (**Supplementary Figure S2C**) or lung (**Supplementary Figure S2D**) at 3 dpi, contrasting with mice that received non-heat inactivated SARS-CoV-2, where most animals had detectable infectious virus at all sites. These data support true SARS-CoV-2 infection of these KI cohorts and not detection of residual inoculum. No infectious SARS-CoV-2 was detected in the brain from any h*ACE2/*h*TMPRSS2* KI or K18 mouse on 2, 4, or 6 dpi (**Figure 2E**). This contrasts with our prior observations of high rates of SARS-CoV-2 neuroinvasion in K18 mice (**Supplementary Figure S3**), where all pre-omicron strains we studied (B.1, alpha, beta, and delta) produce high brain titers in the majority of K18 inoculated mice. Together, these data show that h*ACE2/*h*TMPRSS2* KI mice are susceptible to infection with SARS-CoV-2 omicron and exhibit oropharyngeal and lung tropism and lack of brain infection,similar to K18 mice. The absence of virus detection in the brain of h*ACE2/*h*TMPRSS2* KI is likely due to reduced neuroinvasive capacity of omicron, which shows the same phenotype in K18 mice.

### 3.3 Pulmonary function assessments in SARS-CoV-2 inoculated mice

**Figure 3:**
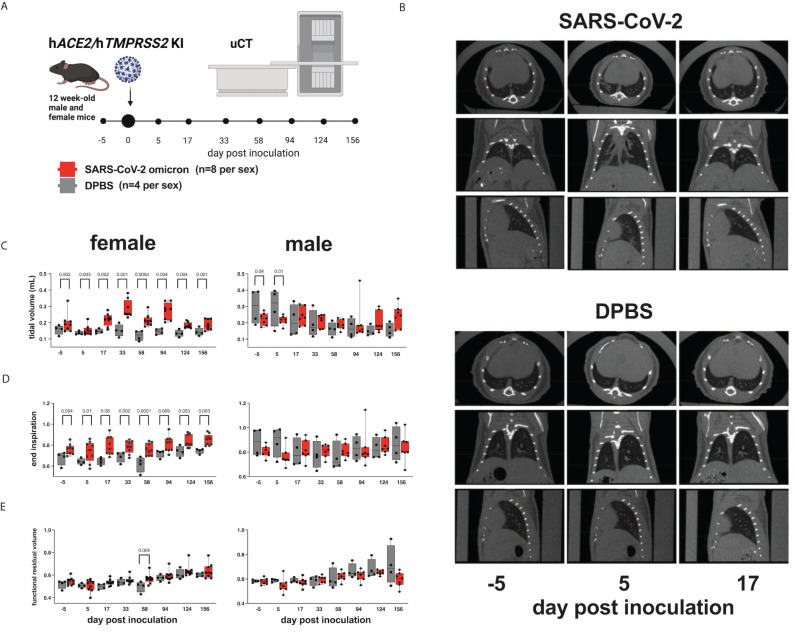
Lung function assessments in h*ACE2/*h*TMPRSS2* KI mice. **(A)** Twelve-week-old male and female h*ACE2*/h*TMPRSS2* KI mice inoculated intranasally with DPBS or 5 log_10_ PFU SARS-CoV-2 B.1.1.529 (omicron) were subjected to µCT to evaluate lung function at -5 (baseline), 5, and 17 dpi and then monthly thereafter to 156 dpi (~6 months). **(B)** Lung µCT images from 2 representative male mice inoculated with SARS-CoV-2 (upper) or DPBS (lower). **(C)** Tidal volume, **(D)** end inspiration, and **(E)** functional residual volume in female or male mice. Statistical designations are based on ANOVA analyses. Each symbol shows a measurement from one mouse, boxes show minimum and maximum measures, lines show means, and error bars show standard deviations. The absence of statistical designations across groups at matched times shows that no statistically significant (p<0.05) differences were measured. Data shown are from one experiment.

To evaluate whether SARS-CoV-2 infection of h*ACE2/*h*TMPRSS2* KI mice produces changes in pulmonary function, we used micro computed tomography (µCT). µCT produces cross-sectional anatomical images enabling detailed internal imaging of the mouse lung. These images can reveal evidence of pulmonary inflammation or changes in functional residual volume, tidal volume, and volume at end inspiration. End inspiration and expiration images from µCT scans were generated over a period of 156 dpi and compared to values 5 days prior to inoculation of SARS-CoV-2 or DPBS (**Figure 3A**). Changes in µCT intensity were detected in some of the male SARS-CoV-2 but not DPBS inoculated h*ACE2/*h*TMPRSS2* mice 5 dpi and resolved by 17 dpi (**Figure 3B**). Female and male mice showed differences in lung function metrics (**Figures 3C-E**). Beginning at -5 dpi and persisting over most times assessed, female SARS-CoV-2 inoculated mice showed significantly higher mean tidal and end inspiration volumes compared with DPBS inoculated mice (ANOVA); functional residual volume was not different most days. Since these differences were detected between treatment groups before SARS-CoV-2 administration, it is not clear whether they are related to viral infection. Male SARS-CoV-2 inoculated mice showed significantly reduced tidal volume compared to DPBS inoculated mice at -5 and 5 dpi (ANOVA); since the -5 dpi differences were detected before virus administration reduced tidal volume was not related to virus infection.

### 3.4 SHIRPA and cardiovascular characteristics in h*ACE2/hTMPRSS2* KI mice

**Figure 4:**
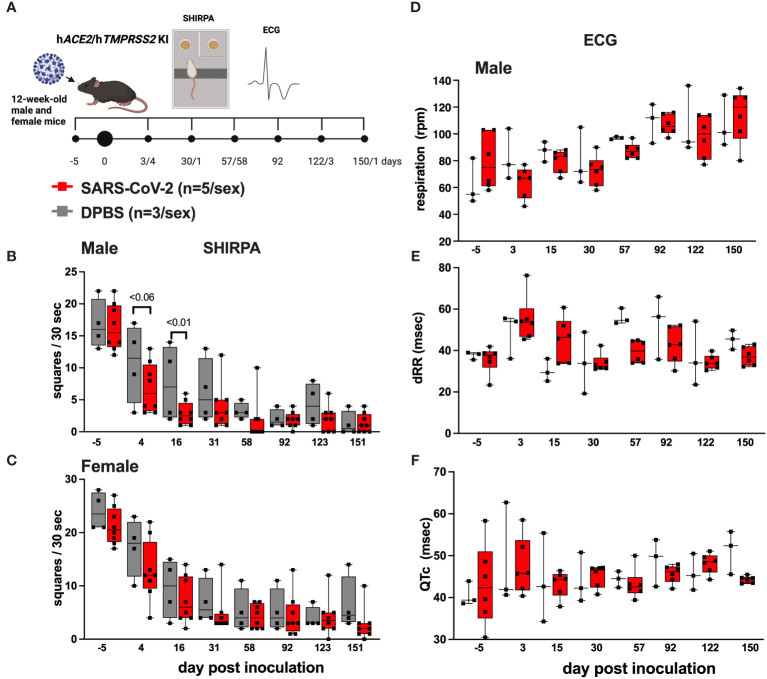
Behavioral and cardiac changes in h*ACE2/*h*TMPRSS2* KI mice as assessed by SHIRPA and ECG. **(A)** Twelve-week-old male and female hACE2/hTMPRSS2 KI mice were inoculated intranasally with DPBS or 5 log_10_ PFU SARS-CoV-2 B.1.1.529 (omicron), and a subset were swabbed and euthanized on or before 6 dpi for virology endpoints (**Figure 2**). The remaining animals were used for behavioral and cardiac assessments. Locomotion was assessed in **(B)** male and **(C)** female mice as the number of squares in a grid that mice occupied in a 30 second period. **(D)** Respiration rates and **(E, F)** cardiac activity markers measured in individual mice. dRR is difference between consecutive R peaks, a marker of heart rate variability, and QTc is QT interval adjusted for heart rate, a marker of ventricular repolarization. Each symbol shows a measurement from one mouse, boxes show minimum and maximum measures, lines show means, and error bars show standard deviations. Statistical designations are based on ANOVA analyses. The absence of statistical designations across groups at other times and in female groups at all times indicates no statistically significant differences were detected. Data shown are from one experiment.

A subset of h*ACE2/*h*TMPRSS2* KI mice inoculated with SARS-CoV-2 or DPBS were evaluated for changes in behavioral and cardiac activity at monthly intervals for up to 151 dpi using SHIRPA and ECG assessment tests (**Figure 4A**). SHIRPA is a standardized set of procedures used to characterize muscle, cerebellar, sensory, and neuropsychiatric function in genetically modified mice. h*ACE2/*h*TMPRSS2* KI mice inoculated with SARS-CoV-2 exhibited no significant differences in reflex responses, gross morphology, or cardiac and respiratory parameters compared to DPBS inoculated mice (**Supplementary Tables S2**, **3**). By contrast, a significant (ANOVA, p<0.01) reduction in locomotion 16 dpi was observed in SARS-CoV-2 inoculated male (**Figure 4B**) but not female (**Figure 4C**) h*ACE2/*h*TMPRSS2* KI mice compared to DPBS inoculated animals. This difference was significant despite the overall decreased ambulation in the test due to habituation to the repetitive nature of the assay. In SARS-CoV-2 inoculated mice of both sexes, no significant changes were detected in cardiorespiratory functions. We measured mild non-significant decreases in respiration rates (**Figure 4D**) and increases in markers of heart rate variability (dRR, **Figure 4E**) and conductivity (QTc **Figure 4F**), compared to DPBS treated animals (male data shown, repeated measures ANOVA, p>0.05; female data was similar). Together these data show that male h*ACE2/*h*TMPRSS2* KI mice experience transient reductions in locomotion after SARS-CoV-2 infection, but that neither sex experiences persistent changes in markers of cardiorespiratory function.

### 3.5 Pulmonary histopathologic changes in h*ACE2/*h*TMPRSS2 KI* mice

**Figure 5:**
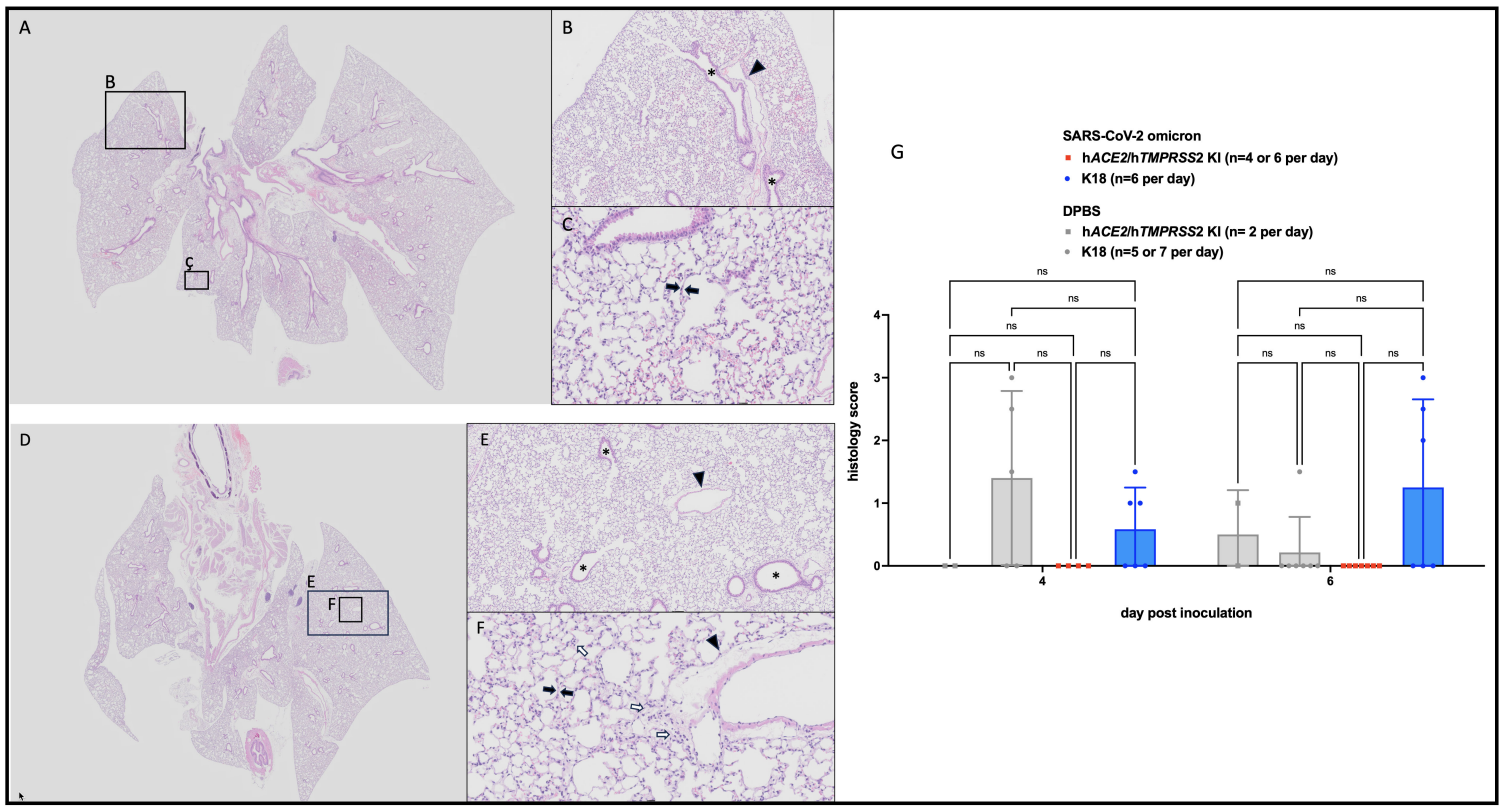
Histopathologic changes in SARS-CoV-2 B.1.1529 (omicron) inoculated h*ACE2/*h*TMPRSS2 KI* and K18 mice. Animals euthanized 6 dpi showed no to minimal histopathologic changes in lungs, mostly having scores of 0. **(A, D)** Low magnification (0.63x) of typical h*ACE2/*h*TMPRSS2 KI* **(A)** and K18 **(D)** lung with homogeneous, aerated parenchyma. **(B, E)** Medium (5x) magnification of indicated regions in A and D, respectively. Alveolar and bronchiolar (*) spaces are clear and free of cellular debris. **(C, F)** High (20x) magnification of the indicated regions in A and D, respectively. Alveolar septa (black arrows) are thin and parenchymal vessels are quiescent (arrowheads). Cellularity is minimally increased but within normal range (white arrows). **(G)** Lung histologic scores. Each symbol shows a measurement from one mouse, boxes show minimum and maximum measures, lines show means, and error bars show standard deviations. Statistical designations are based on ANOVA analyses, ns is not significant at p<0.05.

Lung histopathologic lesions were evaluated in a subset of h*ACE2/*h*TMPRSS2* KI (**Figures 5A-C**) and K18 (**Figures 5D-F**) mice inoculated with SARS-CoV-2 omicron or DPBS and euthanized 6 dpi. Pulmonary histopathology was largely unremarkable for all groups of mice, with most mice scoring 0 (**Figure 5G**). Two DPBS-inoculated mice scored 1 (**Supplementary Figure S4**), indicative of a mild irritating effect due to DPBS. Histopathology scores across any groups were not statistically different (2 way ANOVA with multiple comparisons, p>0.05). These results suggest that infection with SARS-CoV-2 omicron produces similar, negligible histopathologic pulmonary changes 6 dpi in h*ACE2/*h*TMPRSS2* KI and K18 mouse models, with rare individual animal variation.

### 3.6 Re-inoculation of h*ACE2/*h*TMPRSS2* KI mice with SARS-CoV-2

**Figure 6:**
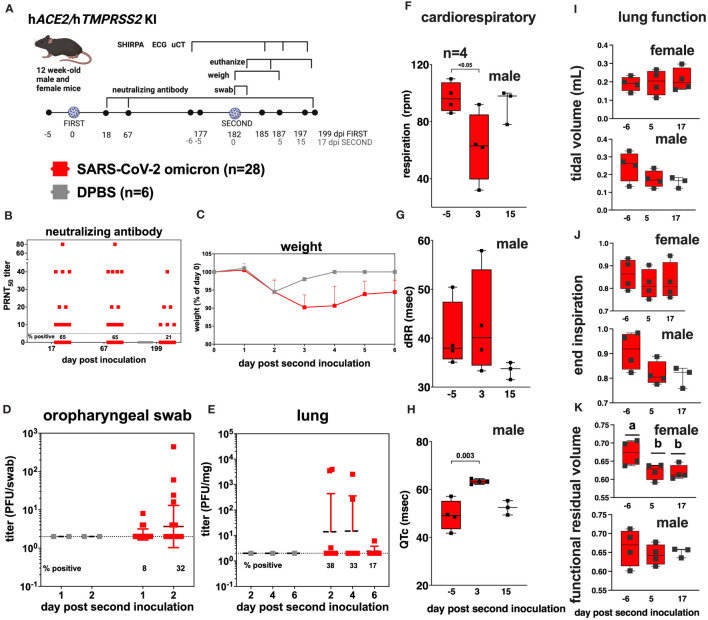
SARS-CoV-2 B.1.1529 (omicron) re-infection kinetics in h*ACE2/*h*TMPRSS2* KI mice. **(A)** Twelve-week-old male and female h*ACE2/*h*TMPRSS2 KI* mice inoculated intranasally with DPBS or 5 log_10_ PFU SARS-CoV-2 B.1.1.529 (omicron) were re-inoculated 182 days later (~6 months) with the same dose and strain of omicron used in the first inoculation. Blood was collected at 18, 67, and 199 dpi, and used for neutralizing antibody assays. Mice were monitored daily and weighed for 6 days, and the oropharyngeal cavity was swabbed 1 and 2 dpi after the second inoculation. A subset of mice were euthanized 2, 4, and 6 dpi after the second inoculation. **(B)** PRNT_50_ titers from mouse serum showing neutralizing antibody titers in SARS-CoV-2 inoculated mice at 3 times post inoculation. Each dot represents the measurement from one mouse. The numbers below the dotted limit of detection lines show the percentage of mice with a detectable PRNT_50_ titer. **(C)** Mean body weight change represented as a percentage of weight at the time of inoculation. Dots represent means and error bars represent standard deviations. A main effect only model two-way ANOVA yielded p<0.0001. Infectious SARS-CoV-2 measured in **(D)** oropharyngeal swabs collected 1 and 2 dpi, and in the **(E)** lung on 2, 4, and 6 dpi, quantified by plaque assay. Symbols represent individual animals, horizontal lines represent geometric mean, and error bars represent geometric standard deviation. The numbers below the dotted limit of detection lines show the percentage of mice with a detectable SARS-CoV-2 titer. A subset of 4 mice of each sex was used for cardiac and lung function assessments. Respiration rates **(F)** and **(G, H)** cardiac activity markers were measured in individual mice, where dRR is heart rate variability and QTc is heart rate. Lung function based on µCT showing **(I)** tidal volume, **(J)** end inspiration, and **(K)** functional residual volume in female and male mice. Statistical designations are based on ANOVA analyses; a versus b indicates p<0.03. The absence of statistical designations across groups indicates no statistically significant differences were detected. Each symbol shows a measurement from one mouse, boxes show minimum and maximum measures, lines show means, and error bars show standard deviations. Data shown are from one experiment.

To simulate repeated SARS-CoV-2 exposure as occurs in humans, we next evaluated SARS-CoV-2 susceptibility and disease manifestations after a second infection. We re-inoculated a subset of SARS-CoV-2 inoculated h*ACE2/*h*TMPRSS2* KI mice with the same strain and dose of omicron used in the first inoculation 6 months after the first inoculation, at 182 dpi, when the mice were 38-weeks-old (**Figure 6A**). Before the second inoculation, 65% (18/28) of serum samples from blood collected 67 dpi from SARS-CoV-2 inoculated mice had detectable PRNT_50_ titers that ranged from 10 to 80 (**Figure 6B**). Re-inoculation of SARS-CoV-2 produced significantly more weight loss to 6 dpi than in DPBS inoculated mice (two-way ANOVA with multiple comparisons, p<0.0001) (**Figure 6C**). Infectious SARS-CoV-2 was detected in oropharyngeal swabs from some mice at 1 and 2 dpi (**Figure 6D**) and in the lungs of some mice 2, 4, and 6 dpi (**Figure 6E**), indicating re-infection. Counterintuitively, seropositivity rates decreased from 65% at 67 dpi to 21% at 199 dpi, where the latter time was 18 days after the second inoculation (**Figure 6B**). Three days after the second inoculation, male mice showed significant reductions in respiration rates (**Figure 6F**, ANOVA, p<0.05), and a trend toward increased heart rate variability (dRR, **Figure 6G**, ANOVA p>0.05), and an increased adjusted QT interval, a marker of ventricular repolarization time (QTc, **Figure 6H**, ANOVA, p<0.003). As observed after the first infection, female mouse respiratory and ECG parameters were not affected (**Supplementary Tables S4**, **S5**). Lung function assessments from µCT showed no significant changes in tidal volume (**Figure 6I**) or end inspiration (**Figure 6J**) for either sex, nor functional residual volume in male SARS-CoV-2 inoculated mice 6 days before compared to 5 or 17 days after the second inoculation (**Figure 6K**). Functional residual volume in female mice was significantly lower at 5 and 17 compared to -6 days post re-inoculation (**Figure 6K**, ANOVA, p<0.03). Together these data show that h*ACE2/*h*TMPRSS2* KI mice are susceptible to a second homologous infection with SARS-CoV-2 6 months after the first infection, characterized by weight loss, respiratory tissue tropism, transient acute changes in cardiac function in male mice, and reduced lung volume in female mice, but that reinoculation does not result in increased neutralizing antibody responses in all mice.

## 4 Discussion

In this study, we generated and characterized a novel mouse model of COVID-19. We expressed both *ACE2* and *TMPRSS2* in dual gene knock-in mice by incorporating the human coding sequence directly into the endogenous mouse loci to create h*ACE2*/h*TMPRSS2* KI mice. These mice express both human genes in respiratory tissues and are susceptible to infection with an omicron strain of SARS-CoV-2. Infection produced transient weight loss and infectious virus detectable in the oropharynx at 1-2 dpi and in the lungs at 2, 4 and 6 dpi. Our data in h*ACE2*/h*TMPRSS2* KI mice are similar to observations from h*ACE2* KI mice (14, 15), although Sun et al. detected interstitial pneumonia, whereas we observed no significant pulmonary histopathologic changes. However, we used omicron whereas both other studies used pre-omicron variants, WA.1, B.1.1.7 (alpha) or B.1.351 (beta). The absence of neuroinvasion by omicron in both h*ACE2*/h*TMPRSS2* KI and K18 mice observed here is consistent with other reports of lower virulence of this variant compared to preceding variants, possibly due to less efficient TMPRSS2 use resulting in lower replication competence as was observed in *ex vivo* human lung explants (27) and reduced fusogenicity in nasal epithelial cell cultures (28). The mild weight loss and near absence of lung histopathologic changes in the h*ACE2*/h*TMPRSS2* KI mice evaluated here contrasts with murine models that transiently express h*ACE2* in the lung via adeno-associated vectors (29, 30), where weight loss was more severe and bronchopneumonia was noted 7 dpi, however, both of those studies were with pre-omicron strains that are generally more virulent than omicron in mouse and hamster models (31–33). Although we were initially surprised that not all h*ACE2*/h*TMPRSS2* KI mice developed detectable neutralizing antibody responses, our studies with heat-inactivated SARS-CoV-2 show that this model and the virus strain and dose used reproducibly establishes a productive infection, despite the lack of seroconversion in all mice. Unfortunately, neither of the other h*ACE2* KI studies (14, 15) evaluated antibody development to allow for a comparison with our results. The absence of neutralizing antibody in all h*ACE2*/h*TMPRSS2* KI mice parallels recent work in K18 mice showing that SARS-CoV-2 infection and vaccine mediated protection can be antibody independent, instead relying on T cell responses (34), which were not a subject of focus in our studies.

To our knowledge, although CT scan is commonly used for defining lung changes in COVID-19 patients (35), no studies to date have used µCT to evaluate lung function in small animal models of COVID-19. One study measured a drug mediated reduction in alveolar damage using µCT in outbred ICR inoculated SARS-CoV-2 mice (36). With uCT and the other cardiovascular metrics used here, we observed that SARS-CoV-2 infection produced visual changes in uCT intensity but no quantitative changes in total lung capacity or other measures of lung function or markers of cardiovascular function in the ensuing 6 months or after a second virus inoculation administered 6 months after the first inoculation. Our observations of reduced ambulation in male but not female mice mirror post-COVID-19 outcomes in people where females experience lower rates of cardiovascular-related hospitalizations after COVID-19 (37). When subjected to a six minute walking exercise followed by pulmonary function tests, males humans also experience higher post-stress respiratory outcomes compared to females (38).

Our study has limitations. The anesthesia and swabbing approach produced weight loss even in DPBS inoculated h*ACE2*/h*TMPRSS2* KI mice, limiting our ability to ascribe weight change to virus treatment. Many of the published studies using similar murine models used pre-omicron strains, limiting direct comparison of outcomes observed in these studies with omicron given that virus strain can impact phenotype. Our studies with h*ACE2*/h*TMPRSS2* KI mice only used omicron and not other SARS-CoV-2 variants. Although we performed power analyses to inform group sizes based on virology metrics prior to starting the project, it is possible the group sizes used were underpowered to detect small differences in lung function or cardiovascular metrics, if they occurred. Our immunology analyses were limited to assessment of neutralizing antibody responses. Future studies should further address immune responses to SARS-CoV-2 infection using the h*ACE2*/h*TMPRSS2* KI model.

Continued development of small animal models of COVID-19 can aid in more fully understanding the pathogenesis of disease caused by SARS-CoV-2. Given that the h*ACE2*/h*TMPRSS2* KI mice were developed on a C57BL/6N genetic background, they can further be employed in cross-breeding experiments with other transgenic mice to enable studies on other COVID-19-associated traits, including diabetes, Alzheimer’s disease, and obesity.

## Data availability statement

The datasets generated and analyzed for this study can be found at https://ucdavis.box.com/s/na5n0mvlhgp4zfx569n5duqmouyo9mo2. Newly generated mouse strains presented in this report (hACE2 KI [RRID:MMRRC_068346-UCD], hTMPRSS2 KI [RRID:MMRRC_032678-UCD], and hACE2/hTMPRSS2 KI [RRID:MMRRC_069727-UCD]) are available from the Mutant Mouse Resource and Research Center (MMRRC) at www.mmrrc.org.

## Ethics statement

The animal study was approved by University of California, Davis Institutional Animal Care and Use Committee. The study was conducted in accordance with the local legislation and institutional requirements.

## Author contributions

HL: Conceptualization, Data curation, Formal analysis, Investigation, Methodology, Visualization, Writing – original draft, Writing – review & editing. TB: Writing – review & editing. AR: Writing – review & editing, Investigation. TW: Investigation, Writing – review & editing. DR: Investigation, Writing – review & editing, Data curation, Formal analysis. MH: Data curation, Investigation, Writing – review & editing, Methodology. AF: Investigation, Writing – review & editing. BW: Investigation, Writing – review & editing, Data curation, Formal analysis, Methodology. JE: Investigation, Writing – review & editing. LL: Investigation, Writing – review & editing, Conceptualization, Data curation, Formal analysis, Methodology, Supervision, Visualization. KL: Conceptualization, Investigation, Supervision, Writing – review & editing, Funding acquisition, Project administration, Resources. LC: Conceptualization, Funding acquisition, Project administration, Resources, Supervision, Writing – review & editing, Data curation, Methodology, Visualization, Writing – original draft.

## Supplementary material

Supplementary Figure 1 Mouse design and production.

Supplementary Figure 2 Heat-inactivated SARS-CoV-2 in *hACE2/hTMPRSS2*.

Supplementary Figure 3 Multiple variant SARS-CoV-2 infection kinetics in K18 mice.

Supplementary Figure 4 Lung histopathology in DPBS-inoculated hACE2/hTMPRSS2 KI mice 6 dpi.

Supplementary Table 1 Primers used to generate KI mice.

Supplementary Table 2 SHIRPA measurements for each mouse.

Supplementary Table 3 ECG measurements for each mouse.

Supplementary Table 4 SHIRPA measurements for each mouse after re-inoculation.

Supplementary Table 5 ECG measurements for each mouse after re-inoculation.
