# Supplementary material for: Establishment and characterization of an hACE2/hTMPRSS2 knock-in mouse model to study SARS-CoV-2
Source: Front Immunol. 2024 Jul 10;15:1428711. doi: 10.3389/fimmu.2024.1428711 (PMC11266032; doi:10.3389/fimmu.2024.1428711)
Supplement: Supplementary Figure 1 — Mouse design and production. [file DataSheet_1.docx]

**Supplemental Figure 1:** **Mouse design and production.** CRISPR based targeting into ES cells followed by blastocyst injection to attain chimeric animals for subsequent backcross to transmit the targeted allele in the germline. (**A**) Location of gRNA cleavage site at the mouse Ace2 locus and replacement with human ACE2 coding sequence (CDS) including the mouse 3’ UTR. (**B**) Genomic copy number validation of ACE2 KI germline heterozygous animal using relative Ct method. (**C**) 5’ and 3’ long range PCR confirmation of the correctly targeted ACE2 KI. (**D**) Location of gRNA cleavage site at the mouse Tmprss2 locus and replacement with human (h) TMPRSS2 coding sequence (CDS) including the mouse 3’ UTR. (**E**) Genomic copy number validation of TMPRSS2 KI germline heterozygous animal (N1) using relative Ct method. (**F**) 5’ and 3’ long range PCR confirmation of the correctly targeted TMPRSS2 KI.

**
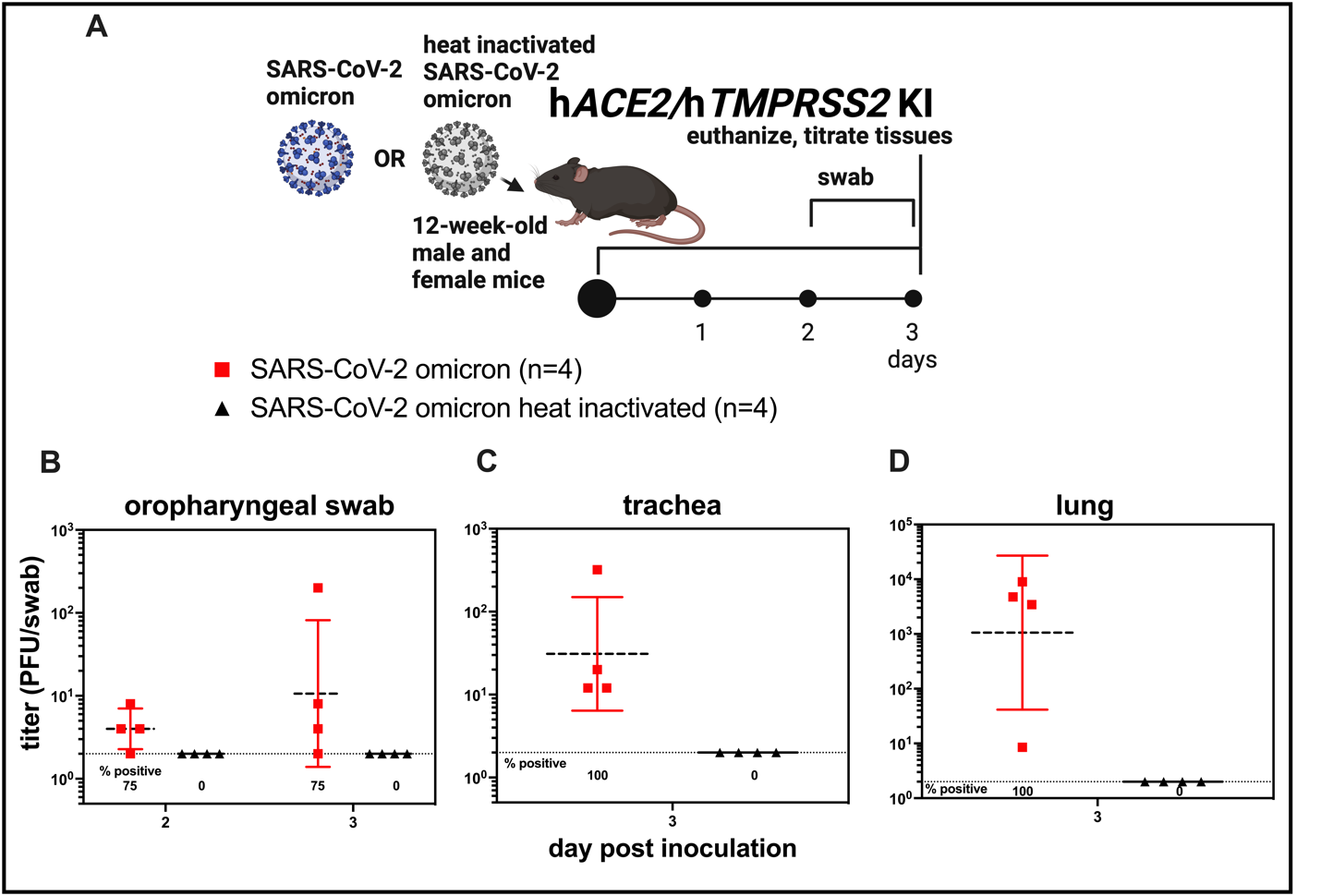
**

**Supplemental Figure 2: Heat-inactivated SARS-CoV-2 in *hACE2/hTMPRSS2* KI mice.** (**A**) Twelve-week-old male and female mice (n=4 per group, 2 males and 2 females) were inoculated intranasally with 5 log_10_ PFU SARS-CoV-2 omicron or with 5 log_10_ PFU SARS-CoV-2 omicron that had been heat inactivated by treatment at 56ºC for 30 minutes. Oropharyngeal swabs were collected 2 and 3 dpi and mice were euthanized 3 dpi. Infectious SARS-CoV-2 measured in the (**B**) oropharyngeal swabs collected 2 and 3 dpi, and in the (**C**) trachea, and (**D**) lung 3 dpi quantified by plaque assay using Vero E6-TMPRSS2-T2A-ACE2 cells. The numbers below the dotted limit of detection lines show the percentage of mice with a detectable SARS-CoV-2 titer. Each value reported shows data from 1 titration.

**
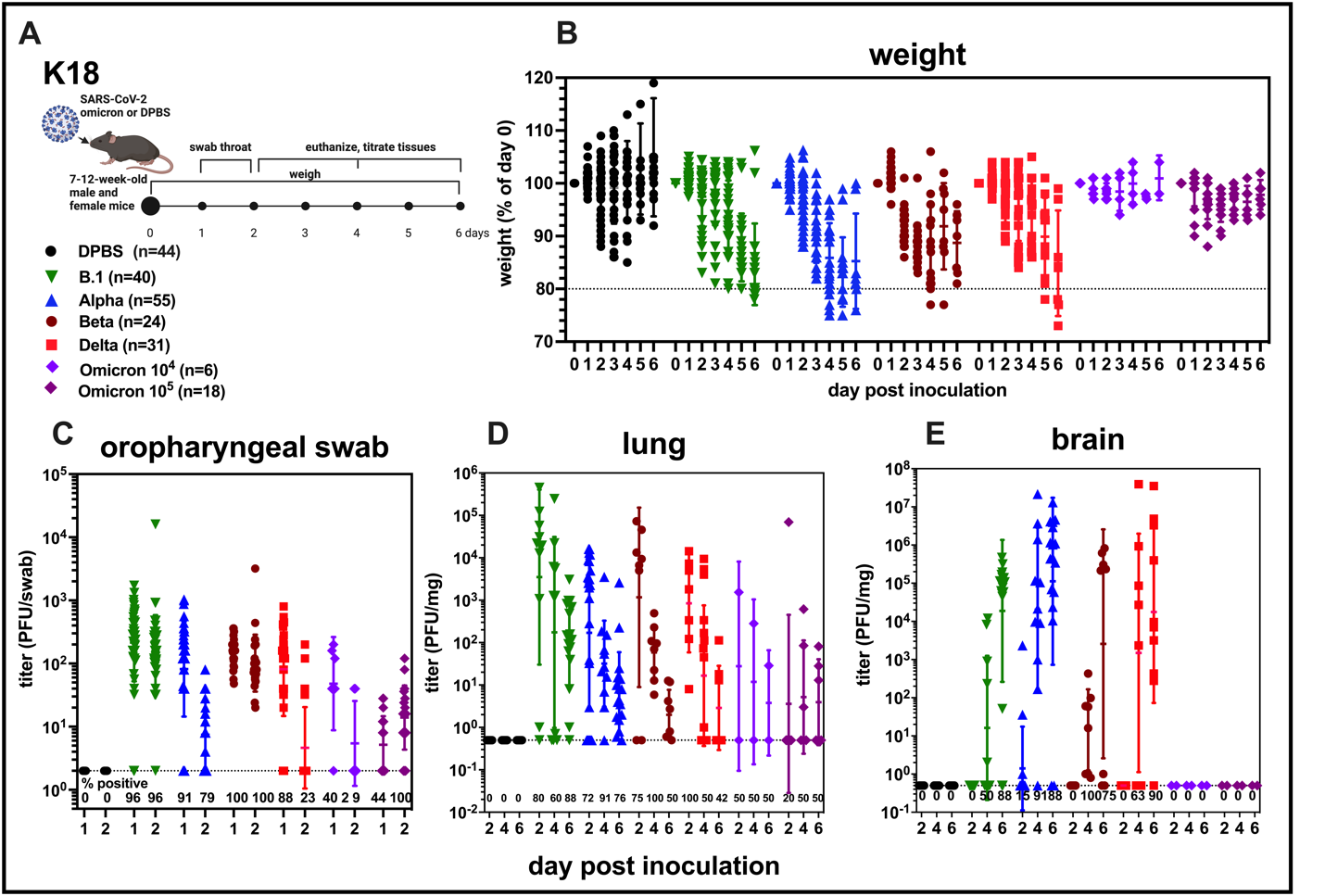
**

**Supplemental Figure 3: Multiple variant SARS-CoV-2 infection kinetics in K18 mice.** (**A**) Seven to 12-week-old male and female mice were inoculated intranasally with DPBS or 4 log_10_ PFU with different SARS-CoV-2 variants A second omicron group was inoculated with 5 log_10_ PFU. Mice were monitored and weighed daily and oropharyngea were swabbed on 1 and 2 dpi. A subset of mice were euthanized 2, 4 and 6 dpi. (**B**) Body weight change represented as a percentage of weight at the time of inoculation. Each symbol represents 1 mouse. Infectious SARS-CoV-2 measured in the (**C**) oropharyngeal swabs collected 1 and 2 dpi, and in the (**D**) lung, and (**E**) brain on 2, 4, and 6 dpi, quantified by plaque assay using Vero-E6 cells except omicron, which was assayed using Vero-E6-TMPRSS2-T2A-ACE2 cells. The numbers below the dotted limit of detection lines show the percentage of mice with a detectable SARS-CoV-2 titer. Each value reported shows data from 1 titration.

**Supplemental Figure 4**: **Lung histopathology in DPBS-inoculated hACE2/hTMPRSS2 KI mice 6 dpi.** Two of 4 mice developed mild lesions with a histopathology score of 1. (**A**) Largely homogenous parenchyma with regional darkening and focal hypercellularity indicated by B and C, respectively. (**B**) Higher magnification of box B illustrating thickened alveolar septa (black arrow) with increased cellularity, predominantly histiocytes. Normal parenchyma immediately adjacent (*) is included for comparison on the right side of the image. (**C**) Higher magnification of box C highlighting perivascular lymphocytic aggregates (arrowhead).
